# Supplementary material for: Altered microRNA Transcriptome in Cultured Human Liver Cells upon Infection with Ebola Virus
Source: Int J Mol Sci. 2021 Apr 6;22(7):3792. doi: 10.3390/ijms22073792 (PMC8038836; doi:10.3390/ijms22073792)
Supplement: Supplementary file 1 [file ijms-22-03792-s001.zip › Supplementary_File/D_ KEGG_Analysis_Results/16-30nt_pathway_Reston-96h-Huh7_vs_Control-96h-Huh7_down.mature_mirna_targets/hsa_EnrichmentScore.pdf]

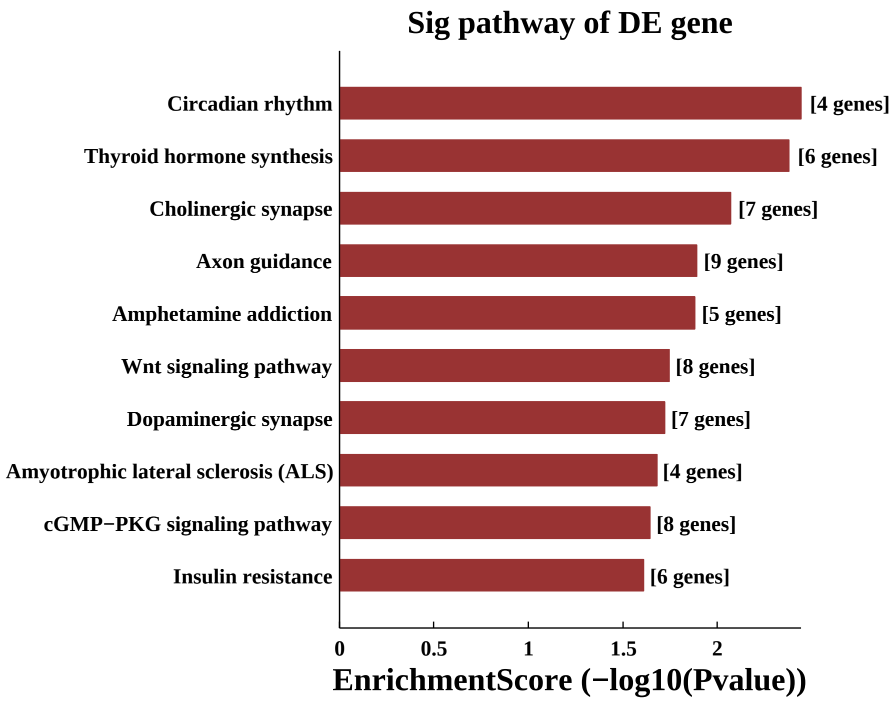
Sig pathway of DE gene
Circadian rhythm
[4 genes]
Thyroid hormone synthesis
[6 genes]
Cholinergic synapse
[7 genes]
Axon guidance
[9 genes]
Amphetamine addiction
[5 genes]
Wnt signaling pathway
[8 genes]
Dopaminergic synapse
[7 genes]
Amyotrophic lateral sclerosis (ALS)
[4 genes]
cGMP−PKG signaling pathway
[8 genes]
Insulin resistance
[6 genes]
0
0.5
1
1.5
2
EnrichmentScore (−log10(Pvalue))
